# Case series: Heinz body formation in 13 multimorbid dogs following metamizole administration

**DOI:** 10.3389/fvets.2023.1183876

**Published:** 2023-07-19

**Authors:** Vera Geisen, Katrin Hartmann, René Dörfelt

**Affiliations:** LMU Small Animal Clinic, Ludwig Maximilian University of Munich, Munich, Germany

**Keywords:** dipyrone, oxidative damage, splenectomy, high MCV, outcome, novalgin

## Abstract

**Background:**

Heinz Body (HB) anemia is a result of oxidative damage and is an uncommon condition in dogs relative to cats. In this retrospective case series, clinical features, laboratory values, concurrent diseases, and outcomes of 13 multimorbid dogs that developed HBs after receiving metamizole are reported.

**Case description:**

Of the 13 dogs in this case series that developed HBs, 10 were older and multimorbid, but the only feature that all the dogs had in common was receiving metamizole. HBs were detected in 7 out of 13 dogs within a few days (3–10 days) after starting metamizole treatment. The metamizole dose was 38–159 mg/kg/day. The highest percentage of HBs detected was 28–95% (mean, 46%). There was no correlation between the percentage of HBs and the daily dose of metamizole. All but 1 dog had mild-to-severe anemia at the time of the highest HB appearance. The number of HBs did not correlate with the hematocrit at that time. In 8/12 dogs, no stress leukogram was present. Approximately half of the dogs with HBs also had evidence of gastrointestinal hemorrhage, which could have masked additional oxidative damage.

**Conclusion:**

In multimorbid dogs that develop regenerative anemia after receiving metamizole, hemolysis due to HB development caused by oxidative damage should be considered as an important differential diagnosis.

## 1. Introduction

Heinz body (HB) formation results from oxidative damage to erythrocytes. HBs are focal accumulations of denatured hemoglobin that are attached to the inner surface of the red blood cell membrane (1–3). In cats, 5–10% of erythrocytes physiologically contain HBs, whereas erythrocytes of dogs physiologically do not contain HBs (4), as cited in (5). This is due to two reasons: first, cats have eight reactive sulfhydryl groups in hemoglobin, while dogs have only four. This makes hemoglobin in cats more susceptible to oxidative damage (6). Furthermore, in contrast to dogs, cats have a non-sinusoidal spleen, which is less effective in eliminating pathological erythrocytes from peripheral blood (7). Several endogenous and exogenous causes of HB have been described in cats. Exogenously, HBs in cats can be induced by oxidant drugs or toxins (methylene blue, repeated administration of propofol, propylene glycol, benzocaine, and methionine) and vegetables such as onions and garlic (3, 8–10). Furthermore, endogenous conditions that cause HB formation include hyperthyroidism, diabetes mellitus, lymphoma, and nonhematological neoplasia (8, 11, 12). Only a few cases of HB anemia have been reported in dogs. The causes in these dogs were intoxication with zinc, methylene blue, acetaminophen, benzocaine, vitamin K_1+3_, naphthalene, phenylhydrazine, ingestion of certain vegetables (onions, chives, garlic), and skunk musk (13–20). Additionally, oxidative damage of erythrocytes indicated by the presence of eccentrocytes was shown in a study in dogs with lymphoma, diabetes mellitus, propofol, non-steroidal anti-inflammatory drugs, and coumarin intoxication prior to treatment with vitamin K (21). HB formation can cause mild to severe anemia. The severity of hemolysis in cats depends on the size and number of HBs as well as the speed and cause of HB formation. Cats with HBs due to propylene glycol have only mild anemia, whereas HBs due to phenylhydrazine intoxication cause more severe hemolysis (3, 11).

Metamizole is a non-opioid analgesic commonly used in dogs in Germany (22). Additionally to the analgesic effect, metamizole acts as an antipyretic and spasmolytic agent (23). So far, there is little evidence that metamizole causes oxidative damage to erythrocytes (24, 25). In humans, metamizole causes a mildly increased risk of gastrointestinal hemorrhage and agranulocytosis; however, HB formation has not yet been described (26, 27). One experimental study in dogs receiving metamizole at a very high dose of 450 mg/kg for 4 weeks intravenously showed a significant increase in HBs, reticulocyte numbers, and bilirubin concentration and a decrease in hematocrit (25). However, this was not observed after subcutaneous application (25). Experimental administration of metamizole for 6 months at a dose of 100 mg/kg caused only a few HBs, whereas doses of 300 mg/kg and 600 mg/kg caused a significant increase in HBs compared to a control group (25). A recent field study showed that metamizole at a standard dose of 40 mg/kg every 8 h caused a significant increase in eccentrocyte concentration, but not in HBs (24).

The present study aims to describe the concurrent diseases, laboratory values, and outcomes in 13 dogs with HB anemia after receiving metamizole.

## 2. Materials and methods

### 2.1. Case selection

The medical records of 13 multimorbid dogs with HB formation that had been presented to the Clinic of Small Animal Medicine, Ludwig-Maximilians-Universitaet (LMU) Munich, Germany, were reviewed. Dogs were included if HBs were detected in the blood smear and if they had received metamizole prior to HB detection. Dogs that had ingested foods, chemicals, or drugs known to cause HBs were excluded.

The protocol for this retrospective study was approved by the Ethics Committee of the Centre for Clinical Veterinary Medicine, LMU Munich, Germany (319-02-27-2022).

### 2.2. Data collection

Data were retrospectively collected from electronic medical records. The data included signalment, history, clinical signs, comorbidities, diagnoses, outcomes, complete blood count results including reticulocytes (Sysmex XT-2000i; Sysmex Deutschland GmbH, Bornbach, Norderstedt, Germany), and percentage of HBs, albumin, total protein, total bilirubin, and creatinine concentrations (Cobas Integra 400 Plus; Roche Diagnostics GmbH, Switzerland).

Brilliant cresyl blue-stained blood smears were evaluated for the presence of HBs in all dogs, and the percentage of HBs was assessed by counting 500 erythrocytes. A manual differential blood count was available in 7/13 dogs.

Regenerative anemia, if present, was classified as hemolysis, hemorrhage, or a combination of both. Hemolysis was considered present if bilirubin increased without evidence of hepatic or posthepatic causes, if there was an increase in bilirubin compared to the previous measurement, or if there was an improvement with treatment (stopping metamizole and/or administration of antioxidative drugs). Hemorrhage was considered present if there was panhypoproteinemia without evidence of other causes for a low total protein level.

### 2.3. Statistical evaluation

Data were analyzed using commercially available statistical software (GraphPad Prism 5; Graph Pad Software Inc.). Data were tested for normality using the D'Agostino and Pearson omnibus normality test and reported as medians (ranges). The correlation of HBs at first appearance and at the highest level with hematologic parameters, dose of metamizole, and duration of administration of metamizole was analyzed using the Spearman correlation test. Statistical significance was set at p < 0.05.

## 3. Results

The median age of the 13 dogs was 10.5 (1.9–14.3) years. Of the 13 dogs, 10 were aged > 10 years (Table 1). The highest mean HBs concentration reported in these 13 dogs was 46% (range, 28–95%). In 11/13 dogs, the highest HB concentration was found at the time point of the initial detection of HBs. Most of the dogs that developed HBs were multimorbid and were treated with multiple drugs (Table 1). However, the only feature that all dogs had in common was that they received metamizole. The median duration of metamizole treatment in the 10 dogs, for which the exact treatment duration was known, was 12 (3–35) days (Table 1). The dose of metamizole administered varied from 38 to 159 mg/kg/day and was divided into two or three doses. There was neither a correlation between the metamizole dose and the number of HBs (*p* = 0.419) nor between the duration of metamizole administration and the number of HBs (*p* = 0.890).

**Table 1:** Signalment, problems, causes of anemia, and hematocrit at the time of highest Heinz Bodies (HBs) numbers, highest HBs (percentage), dose/application of metamizole, days after metamizole of which HB appeared, treatment, and outcome in 13 dogs with HB formation after receiving metamizole.

**Case number**	**Age (years)**	**Weight (in kg)**	**Breed**	**Sex**	**Problems/ diagnoses **	**Cause/s of anemia **	**Hematocrit (time of highest HBs) (reference range: 0.35-0.58 l/l) **	**Highest HBs (%) **	**Dose/ application metamizole **	**HB appearance in days after metamizole **	**Other drugs **	**Treatment of HB **	**Outcome**
1	11.7	9.1	Labradoodle	FS	hyperadrenocorticism, history of splenectomy	hemolysis	0.31	32	50 mg/kg TID p.o.	3	omeprazol trilostane	SAME p. o. 20 mg/kg SID	R
2	14.3	20.7	Belgian shepherd dog	FS	CKD stage 3, hypertensive, proteinuric, AV-block grade 3, pacemaker, orthopedic pain	hemolysis, bleeding	0.35	46	12,5 + 25 mg/kg p.o.	un-known	benazeprile ephedrine pimobendane sotalole pregabalin	SAME p. o., 20 mg/kg BID	R
3	5.5	4.7	Deer Pinscher	MN	glomerulopathy, suspected pancreatitis, hypoadrenocorticism, DIC	hemolysis	0.15	60	53 mg/kg TID p.o.	9	prednisolone omeprazole sucralfate marbofloxacin buprenorphine maropitant legaphyton ursochol	ACC	E day 2
4	10.4	34.8	Dobermann Pinscher	FS	lymphoma multicentric, suspected pancreatitis, splenic mass	hemolysis	0.21	41	50 mg/kg TID i.v.	6	maropitant amoxiclav pregabalin prednisolone	SAME p. o., 40 mg/kg BID	E day 2
5	13.2	37.1	Labrador	M	diabetes mellitus, splenic mass, seizures, enlarged lymph nodes, testicular mass	hemolysis? bleeding	0.25	41	27mg/kg TID p.o.	35	phenobarbital caninsulin omeprazole sucralfate	SAME p. o., 40 mg/kg BID for 2 days, than 20 mg/kg BID	E day 8
6	11.4	29.6	Border Collie	MN	cauda equina compression syndrome, artificial hip, hypothyroidism, severe mitral valve endocardiosis	hemolysis	0.32	90	50 mg/kg TID p.o.	several months	pimobendane thyroxin, pancreatin, gabapentin, tramadol	SAME p. o., 40 mg/kg BID for 1 day, than 20 mg/kg BID	R
7	7.7	8.6	French bulldog	FS	glomerulopathy, azotemia	hemolysis, bleeding	0.31	32	30 mg/kg BID p.o.	8	telmisartan omeprazole pregabalin calcium carbonate lanthan carbonate	SAME p. o., 20 mg/kg BID	R
8	10.5	13.5	Whippet	M	hyperadrenocorticism, azotemia, hypertension, mitral valve endocardiosis	hemolysis? bleeding	0.22	30	38 mg/kg TID p.o.	17	trilostane	SAME p. o., 15 mg/kg SID	E day 17
9	10.5	3.6	Chihuahua	MN	herniated disc, splenic mass, pain	hemolysis	0.20	28	46 mg/kg TID p.o.	10	methocarbamol gabapentin carprofen	ACC	R
10	13.9	10.5	Dachshund	F	azotemia, history of pyometra, suspected hyperadrenocorticism, mass spleen	hemolysis	0.26	28	36 mg/kg TID p.o.	8	amoxicillin/ clavulanic acid, ursochol gabapentin	ACC	E day 4
11	10.3	7.5	Mixed breed	FS	DKA, pancreatitis, CKD, hypophosphatemia	hemolysis, bleeding	0.08	95	50 mg/kg TID i.v.	6	insulin, amoxicillin/ clavulanic acid, omeprazole, buprenorphine	none	E day 1
12	1.9	8.5	Kooikerhondje	F	AKI post anesthesia	hemolysis	0.34	45	30 mg/kg TID p.o.	18	maropitant ondansetrone metoclopramide omeprazole sucralfate calcium carbonate amoxicillin/ clavulanic acid	ACC	R
13	13.3	31.3	Mixed breed	FS	multiple orthopedic problems, history of splenectomy	hemolysis, bleeding	0.27	30	32 mg/kg TID p.o.	weeks un-known	sucralfate silymarin	owners refused	R

ACC, acetylcysteine; AKI, acute kidney injury; BID, twice a day; CKD, chronic kidney disease; DIC, disseminated intravascular coagulopathy; DKA, diabetic ketoacidosis; E, euthanasia; F, female; hemolysis?, no clear evidence of hemolysis; HB, Heinz body; M, male; N, neutered; p, oral; R, recovered; SAME, S-adenosylmethionine; S, spayed; TID, three times a day.

All but 1 dog had mild-to-severe anemia at the time of the highest HB number (Table 2). This dog (Dog 2) did not have anemia, but the hematocrit dropped by 10% compared to the previous value, which was analyzed 4 months prior. There was no correlation between HB concentration and anemia severity (p = 0.929). Reticulocytes were measured in 12/13 dogs and were elevated in all 12 of them, although, in 2 dogs (Dog 2 and Dog 4), they were only mildly elevated. In addition, the MCV was elevated in 12/13 dogs.

**Table 2:** Complete blood count, bilirubin, total protein, albumin, and creatinine concentrations (with reference ranges in parentheses) in 13 dogs with Heinz Body (HB) formation after receiving metamizole at the time when HBs were first visible.

**Case number**	**WBC (5–16 × 10^∧^9/l) **	**RBC (5.5–9.3 × 10^∧^12/l) **	**HGB (7.45–12.5 mmol/l) **	**HCT (0.35–0.58 l/l)**	**MCV (58–72 fL) **	**PLT (150–500 × 10^∧^9/l) **	**Neutrophiles (3–9 × 10^∧^9/l) **	**Lymphocytes (1–3.6 × 10^∧^9/l) **	**Monocytes (0.04–0.5 × 10^∧^9/l) **	**Eosinophils (0.04–0.6 × 10^∧^9/l) **	**Basophils (0–0.04 × 10^∧^9/l) **	**Reticulocytes (< 60 × 10^∧^9/l) **	**Total bilirubin (0–5.26 μmol/l) **	**Total protein (55.5–77.6 g/l) **	**Albumin (31.3–43 g/l) **	**Creatinine (44–125 μmol/l) **
1	21.59	4.51	6.9	0.33	73.2	676	12.41	7.19	1.35	0.59	0.05	232.7	2.2	61.6	45.3	56
2	7.71	4.66	6.9	0.36	76.4	479	5.45	1.26	0.41	0.59	0	63.4	1.4	55.7	28.5	210
3	16.76	1.94	3.0	0.16	82.0	51	14.04	1.71	0.70	0.31	0	96.8	1.5	41.2	18.8	91
4	7.40	2.54	3.5	0.19	73.2	41	3.20	1.85	2.30	0.04	0.01	61.2	12.8	46.8	31.8	66
5	10.76	3.20	5.0	0.26	80.9	628	7.87	1.78	0.75	0.35	0.01	185.6	1.6	56.3	33.3	94
6	9.04	4.14	6.3	0.32	77.5	666	6.57	1.84	0.31	0.32	0	192.0	n.d.	n.d.	n.d.	n.d.
7	6.68	4.17	6.4	0.31	74.3	252	5.67	0.73	0.19	0.09	0	144.7	2.4	46.2	23.9	254
8	5.35	2.64	4.1	0.21	82.6	644	4.61	0.38	0.25	0.11	0	85.0	1.7	44.3	30.7	268
9	9.33	2.52	4.0	0.20	82.9	166	6.39	1.75	1.07	0.10	0.02	164.1	0.9	72.0	33.1	n.d.
10	30.66	3.30	5.0	0.26	78.8	733	26.8	2.18	1.46	0.18	0.04	351.4	8	57.9	32.1	240
11	50.22	1.05	1.4	0.08	74.3	236	n.d.	n.d.	n.d.	n.d.	n.d.	n.d.	9.5	45.0	24.2	174
12	10.96	4.37	6.3	0.31	72.8	418	7.49	2.17	0.90	0.39	1.00	208.4	4.7	48.1	28.6	400
13	9.62	4.08	5.7	0.27	66.7	1008	7.35	1.32	0.41	0.54	0	108.1	3.6	58.1	35.7	102
median	9.62	3.32	5.0	0.26	76.4	479	6.6	1.77	0.73	0.32	0.01	154.4	2.2	56.3	32.1	225.0
range	5.35–50.22	1.05–4.66	1.4–6.9	0.08–0.36	66.7–82.9	41–1008	3.2–26.8	0.38–7.19	0.19–2.30	0.04–0.59	0.00–1.00	61.2–351.4	0.9–12.8	41.2–72.0	18.8–45.3	56.0–400.0

n.d., not done; HCT, hematocrit; HGB, hemoglobin; MCV, mean corpuscular volume; PLT, platelets; RBC, red blood cells; WBC, white blood cells.

Leukocytes were within the reference range in 9/13 dogs and neutrophils were within the reference range in 8/12 dogs, for which a differential blood count was performed (Table 2). Of the four dogs with leukocytosis, two had hyperadrenocorticism (Dogs 1 and 10).

Thrombocytosis was detected in 6/13 dogs (Dog 1, Dog 5, Dog 6, Dog 8, Dog 10, and Dog 13) (Table 2). A total of 3 dogs with thrombocytosis had hyperadrenocorticism (Dog 1, Dog 8, and Dog 10), and 2 had undergone a prior splenectomy (Dog 1 and Dog 13) (Table 1). In 4/7 dogs with thrombocytosis, there was evidence of hemorrhage (Dog 5, Dog 8, and Dog 13) as an (additional) potential cause of anemia.

A manual differential blood count was available for 7/13 dogs. A high number of eccentrocytes was reported in only 1 dog (Dog 9).

Bilirubin concentration levels were measured in 12/13 dogs. They were elevated at the time of the highest HBs (or shortly after) in 3/12 dogs (Table 2). In 5/12 dogs, concentration increased but remained within the reference range (Dog 1, Dog 2, Dog 7, Dog 9, Dog 12). Of the 13 dogs, 3 (Dog 6, Dog 12, and Dog 13) were classified as having hemolysis by a drop in HBs and an increase in hematocrit following discontinuation of metamizole and/or application of antioxidants. In 2 of these dogs (Dogs 12 and Dog 13), the bilirubin concentration was in the upper reference range (> 3.5 μmol/L) at the time of the highest HBs. In the third dog (Dog 6), a serum profile was not obtained when HBs were detected. In 2 dogs (Dogs 5 and Dog 8) with HBs, there was no clear evidence of hemolysis.

Albumin and total protein levels were decreased in 6/12 dogs (Table 2). In 1 dog (Dog 3), there was a loss of albumin and total protein in the kidney (protein-losing nephropathy) and gut (hypoadrenocorticism). In another dog (Dog 12), there was mild panhypoproteinemia, but albumin and total protein concentration did not decrease parallel to the decrease in hematocrit, whereas bilirubin concentration increased. Only 1 dog (Dog 4) had a decrease in total protein concentration, while the albumin concentration remained within the reference range; bilirubin concentration was above the reference range. Anemia in the three cases mentioned above was therefore classified as hemolysis. In the remaining three cases of panhypoproteinemia (Dog 7, Dog 8, and Dog 11) and in one case of hypoalbuminemia (Dog 2), the cause of anemia was likely a combination of hemorrhage and hemolysis. Therefore, regenerative anemia was classified as hemolysis in 7/13 dogs, as a combination of hemorrhage and hemolysis in 4/13 dogs, and in 2/13 dogs, it was not clear whether there was only hemorrhage or additional hemolysis (Table 1).

Most of the dogs were multimorbid (Table 1). Of the 11 dogs in which creatinine was measured, 6 showed mild-to-moderate azotemia. Of the 13 patients, 3 had concurrent hyperadrenocorticism and 2/13 had a previous splenectomy. In 4 of the remaining 9 dogs in which ultrasound was performed, there was a small mass in the spleen. Of the 13 dogs, 6 were euthanized within 2 to 8 days.

**Figure 1:**
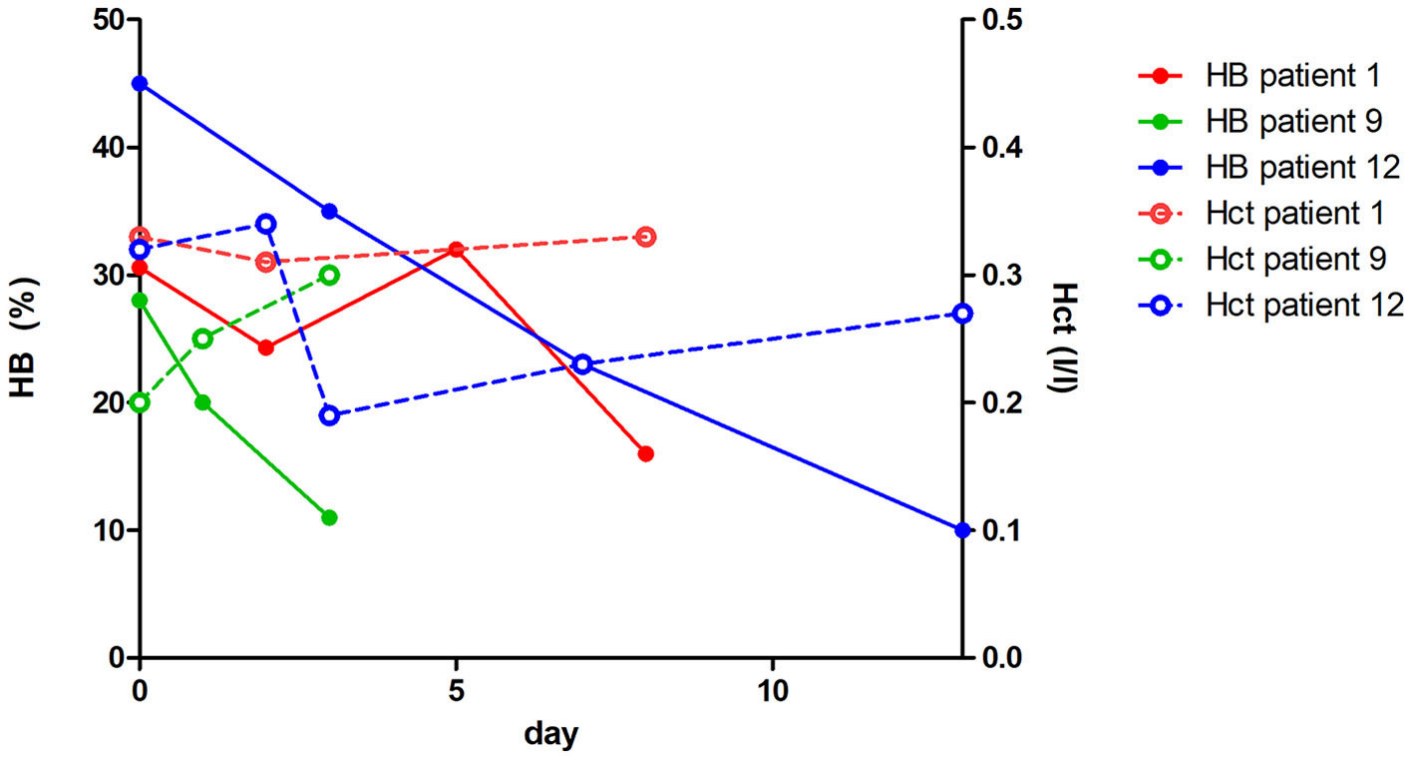
Three dogs (Dog 1, Dog 9, and Dog 12) in which hematocrit and Heinz bodies were measured at least three times. HB, heinz body; Hct, hematocrit.

In all dogs, metamizole administration was stopped after HBs were detected. Of the 13 dogs, 11 were treated with antioxidants, 7 with S-adenosylmethionine (SAME), and 4 with acetylcysteine (ACC) (Table 1). A follow-up of hematocrit was available for 9 dogs; in 3 dogs, hematocrit and HBs were measured at least three times (Figure 1, Supplementary Table 1). There was an increase in hematocrit in 5 dogs, a decrease in 3 dogs, and a stable hematocrit in 1 dog within the following days after the highest HBs were detected. In 8/9 dogs with at least two measurements of HBs, there was an immediate decrease in HB numbers after discontinuing metamizole (Supplementary Table 1). A total of seven dogs received antioxidant therapy (Table 1).

## 4. Discussion

In this case series, 13 multimorbid dogs with HB formation following metamizole administration were described. Of the 13 dogs in this case series, 12 had regenerative anemia. Since most of the dogs were older and multimorbid and all of them received metamizole, which is known to increase the risk of gastrointestinal hemorrhage in humans, the first differential diagnosis for the regenerative anemia in these dogs was a gastrointestinal hemorrhage (26). However, all dogs had HBs, and except for 2 dogs, all had additional evidence of hemolysis. A previous study showed that metamizole could induce mild oxidative damage by the production of eccentrocytes in dogs, but HBs were not detected at the recommended dose (24). In an older experimental study, only very high doses (300 and 600 mg/kg) of metamizole administered for a long period (26 weeks) caused a statistically significant increase in HBs compared to the control group (25). In a study of metamizole in cats, no oxidative damage to cat erythrocytes was detected (28). The mechanism by which metamizole causes HBs is unknown. Lieser et al. (24) showed a significant increase in eccentrocytes and reticulocytes and a decrease in hemoglobin in dogs receiving a therapeutic dose of metamizole compared with dogs of control groups receiving meloxicam or carprofen. Although eccentrocytes are indicative of oxidative damage, glutathione peroxidase and superoxide dismutase, which are important enzymatic antioxidative defense systems in erythrocytes, did not differ between these groups (24). All the dogs in the present case series had at least one underlying problem/disease. Azotemia was observed in 6 dogs. In one study, there was 1 dog with chronic kidney disease and eccentrocytes (21), and in another study evaluating the oxidative stress on erythrocytes in 30 dogs with chronic kidney disease (IRIS stages 3 and 4), no dog had eccentrocytes or HBs. This study also showed a compensatory response of superoxide dismutase and sustained erythrocytic concentration of reduced glutathione in dogs with chronic kidney disease and anemia, despite evidence of increased systemic oxidative stress (29). Therefore, it is unlikely that in the present case series, oxidative damage was due to chronic kidney disease. Of the 13 dogs, 2 (Dog 1 and Dog 13) had undergone a previous splenectomy. In humans, the spleen is important for removing abnormal erythrocytes, including erythrocytes with HBs from the peripheral blood (30). Splenectomy is likely an important factor because erythrocytes with HBs are removed more slowly from the circulation, and HB values are therefore higher. In this case series, 2 dogs (Dog 5 and Dog 11) had diabetes mellitus, and 1 (Dog 3) had diabetic ketoacidosis. Studies have shown that diabetic ketoacidosis can cause oxidative damage to erythrocytes in cats and dogs (11, 12, 20, 21). Additionally, some dogs in the present case series had inflammatory diseases (pancreatitis [Dog 3, Dog 4, and Dog 11] and pyometra [Dog 10]), which could contribute to oxidative damage, and 1 dog (Dog 4) had lymphoma, which could also cause oxidative damage (20, 21, 31). Since there was no correlation between the number of HBs and the dose of metamizole and because many dogs in this case series had multiple diseases/problems, the oxidative damage to erythrocytes could have been multifactorial and not only related to oxidative damage secondary to metamizole administration, at least in some dogs.

There was no correlation between the concentration of HBs and the hematocrit. In cats, the severity of hemolysis depends on the number and size of HBs as well as the speed and cause of HB formation. Cats with HBs due to propylene glycol have only mild anemia, whereas HBs due to phenylhydrazine intoxication cause more severe hemolysis (3, 11). In dogs, there are no data available on whether hematocrit correlates with the concentration of HBs. Since hemolysis was the sole cause of anemia in only 7/13 dogs and additional bleeding was present in some dogs, it was difficult to evaluate a correlation in this case series. However, 1 dog with 90% of erythrocytes containing HBs (Dog 6) had only mild anemia, with a hematocrit of 32%. All 12 dogs in which reticulocytes were detected also had reticulocytosis. Additionally, MCV was elevated in 12/13 dogs. In a retrospective study evaluating the diagnostic value of erythrocyte indices in differentiating between regenerative and non-regenerative anemia, only 16.8% (240/1,426) of dogs showed macrocytic anemia (32). Therefore, in the present case series, macrocytosis was present in a higher percentage of dogs with regenerative anemia, as described previously. Interestingly, HBs are known to interfere with automated hematological analyses. They can cause erroneously high total white blood cell counts and an increase in MCHC and MCH, but there is no evidence of artificial elevation of MCV (33–35). Additionally, HBs can artificially increase reticulocyte counts because of their increased fluorescence (36). However, in the presented cases, reticulocytosis and macrocytosis were probably caused by the regenerative nature of anemia.

Although the patients had comorbidities and a stress leukogram would have been suspected, 8/12 dogs, in which a differential blood count had been performed, had neutrophils within the reference range, 4 of them even in the lower half of the reference range. Most dogs with HB formation secondary to vitamin K_1_ treatment also have neutrophilia (19). In a toxicity study in dogs, metamizole caused a decrease in the white blood cell count at a dose of 450 mg/kg administered for 4 weeks (25). In humans, agranulocytosis and neutropenia are well-documented adverse events of metamizole (37, 38). However, the underlying mechanisms are not well-understood. It has been assumed that immunological mechanisms, including T-cell-mediated destruction of neutrophils, are involved (39). However, some criteria, such as the rapid onset within hours after the first dose in some patients, are compatible with direct toxicity of neutrophil precursors in the bone marrow (27). Thus, in the present case series, the missing stress leukogram was suspected to be a consequence of metamizole treatment.

A high number of eccentrocytes was detected in only 1 dog (Dog 3). Eccentrocytes are caused by oxidative damage to the membrane and cytoskeleton of erythrocytes, while HBs are caused by damage to hemoglobin (21). In a previous study, it was hypothesized that the first sign of oxidative damage to erythrocytes in cats is HBs, whereas, in dogs, eccentrocytes are present first due to oxidative damage to the erythrocyte membrane (21). Thus, it is possible that the dogs in the present study had oxidative damage in an advanced stage or a severe form. However, in a study examining hematological changes in dogs after garlic administration, the number of eccentrocytes increased more severely than the number of HBs and, thus, eccentrocytes were suspected to be the cause of hemolysis (40). In the present study, the presence of eccentrocytes was probably underestimated because not all dogs underwent a manual evaluation of a blood smear; therefore, the true number of dogs with eccentrocytes could not be determined.

A limitation of this study is that manual differential counts and evaluation of blood smears were not available for all dogs. Therefore, it is likely that eccentrocytes were missed in some patients. Additionally, owing to the retrospective nature of this case series, there was limited follow-up and the course of anemia was known in only a few cases. Furthermore, some dogs in this case series had at least one other problem/disease that could have caused HB formation; therefore, oxidative damage to erythrocytes might have been multifactorial in those dogs and not only related to oxidative damage secondary to metamizole administration.

In conclusion, in multimorbid dogs that develop regenerative anemia after receiving metamizole, HBs leading to hemolysis due to oxidative damage can occur. Thus, multimorbid dogs, especially those with splenectomy or splenic masses, seem to be predisposed to develop HB anemia after metamizole application, and in those dogs, metamizole treatment should only be performed with frequent controls of CBC.

## Data availability statement

The original contributions presented in the study are included in the article/Supplementary material, further inquiries can be directed to the corresponding author.

## Ethics statement

The protocol of this retrospective study was approved by the Ethics Committee of the Centre for Clinical Veterinary Medicine, LMU Munich (319-02-27-2022).

## Author contributions

VG, KH, and RD contributed to the conception and design of this study. RD performed the statistical analyses. VG wrote the first draft of the manuscript. All authors contributed to the manuscript revision and have read and approved the submitted version.
