# Supplementary material for: Case series: Heinz body formation in 13 multimorbid dogs following metamizole administration
Source: Front Vet Sci. 2023 Jul 19;10:1183876. doi: 10.3389/fvets.2023.1183876 (PMC10394240; doi:10.3389/fvets.2023.1183876)
Supplement: Supplementary file 1 [file Table_1.docx]

Supplemental Table 1: Course of hematocrit and HBs in 13 dogs with Heinz body (HB) formation after receiving metamizole. Day 0 is the day HBs were detected for the first time. Days with “-” are the number of days prior to the first detection of HBs.

| **case number** | **development** | | |
| --- | --- | --- | --- |
|  | **day** | **HB (%)** | **HCT (l/l)** |
| 1 | -3 | n. d. | 0.41 |
|  | 0 | 31 | 0.31 |
|  | 1 | 24 | 0.33 |
|  | 4 | 32 | 0.32 |
|  | 7 | 12 | 0.33 |
|  | 22 | 6 | 0.35 |
|  | 35 | 1 | 0.41 |
| 2 | -120 | n. d. | 0.46 |
|  | 0 | 46 | 0.36 |
|  | 6 | 18 | 0.36 |
| 3 | -2 | n. d. | 0.17 |
|  | 0 | 49 | 0.16 |
|  | 1 | 60 | 0.15 |
| 4 | -2 | n.d. | 0.37 |
|  | -1 | n.d. | 0.26 |
|  | 0 | 41 | 0.21 |
| 5 | 0 | 41 | 0.25 |
| 6 | -24 | n. d. | 0.42 |
|  | -22 | n. d. | 0.35 |
|  | 0 | 90 | 0.32 |
|  | 8 | n.d. | 0.35 |
|  | 22 | 0 | 0.41 |
| 7 | -23 | n.d. | 0.43 |
|  | 0 | 32 | 0.31 |
|  | 8 | 4 | 0.29 |
| 8 | 0 | 30 | 0.22 |
|  | 4 | 19 | 0.22 |
| 9 | 0 | 28 | 0.2 |
|  | 1 | 20 | 0.25 |
|  | 3 | 11 | 0.30 |
| 10 | 0 | 28 | 0.26 |
|  | 1 | n.d. | 0.21 |
|  | 2 | n.d. | 0.19 |
| 11 | 0 | 95 | 0.08 |
| 12 | 0 | 45 | 0.32 |
|  | 3 | 35 | 0.19 |
|  | 7 | 20 | 0.23 |
|  | 13 | 10 | 0.27 |
| 13 | 0 | 30 | 0.27 |
|  | 14 | 14 | 0.36 |

n.d. = not done, HCT = hematocrit
